# Cyclooxygenase‐2 expression correlates with development, progression, metastasis, and prognosis of osteosarcoma: a meta‐analysis and trial sequential analysis

**DOI:** 10.1002/2211-5463.12560

**Published:** 2019-01-07

**Authors:** Shengqun Wang, Hongwei Gao, Jianlin Zuo, Zhongli Gao

**Affiliations:** ^1^ Orthopaedics China‐Japan Union Hospital of Jilin University China; ^2^ Orthopaedics The Affiliated Hospital to Changchun University of Chinese Medicine Jilin China

**Keywords:** clinical features, cyclooxygenase‐2, expression, osteosarcoma, prognosis

## Abstract

Cyclooxygenase‐2 (COX‐2), a key enzyme in arachidonic acid metabolism, is involved in several cancers, including osteosarcoma. The prognostic significance of COX‐2 in osteosarcoma remains controversial. This study was to analyze the potential clinical and prognostic effects of COX‐2 protein expression in patients with osteosarcoma. Eligible articles were searched via online databases. The combined odds ratios (ORs) or hazard ratios (HRs) with their 95% confidence intervals (95% CIs) were calculated using the random‐effects model. Trial sequential analysis (TSA) was applied to analyze the required information size and determine the reliability of the evidence. Twenty‐three studies on COX‐2 expression were identified, which included a total of 1084 patients with malignant osteosarcoma and 247 patients with benign osteochondroma. COX‐2 protein expression in osteosarcoma was higher than in benign osteochondroma (OR = 7.66, *P *<* *0.001). COX‐2 expression was not correlated with age, gender, tumor location, cancer histology, or necrosis (*P *>* *0.1), but was significantly associated with tumor grade (high grade vs. low grade: OR = 4.81, *P *<* *0.001), clinical stage (stage 3–4 vs. stage 1–2: OR = 4.89, *P *<* *0.001), and metastasis (yes vs. no: OR = 3.53, *P *<* *0.001). Based on TSA results, we suggest that additional studies are not required to examine osteosarcoma vs. benign osteochondroma, tumor grade, clinical stage, or metastasis. No heterogeneity was observed in these analyses. COX‐2 expression is linked to poor prognosis in metastasis‐free survival, overall survival, and relapse‐free survival, as indicated by multivariate analysis. Therefore, the expression of COX‐2 may correlate with the development, progression, metastasis, and poor prognosis of osteosarcoma.

**Abbreviations**
95% CI: 95% confidence interval
APIS: a priori anticipated information size
COX‐2: cyclooxygenase‐2
HR: hazard ratio
IHC: immunohistochemistry
OR: odds ratio
PRISMA: Preferred Reporting Items for Systematic Reviews and Meta‐Analyses
PTGS: prostaglandin‐endoperoxide synthase
RRR: relative risk reduction
TSA: trial sequential analysis

Osteosarcoma is the most frequent primary bone sarcoma that occurs mainly in children and adolescents 1. Approximately 4.4 per million are diagnosed with osteosarcoma every year 1, 2. Osteogenic sarcoma consists of several main histotypes: osteoblastic, chondroblastic, and fibroblastic osteosarcoma 3. Osteogenic osteosarcoma is the most common pathological subtype 3. Although the biology of osteosarcoma is complex, treatment options have not significantly changed over the past several decades 4. Osteosarcoma can be caused by other types of cancer and some environmental factors such as viruses and radiation 5. This rare disease is treated by advanced surgery and adjuvant/neoadjuvant chemotherapy 6, 7. Current treatment for osteosarcoma achieves a 5‐year survival rate of 20% to 30% in metastatic or recurrent osteosarcoma patients 8. When diagnosed, 40% of metastasis occurs in osteosarcoma patients at an advanced stage 9.

Increasing evidence shows that molecular mechanisms are correlated with the development, progression, and prognosis of osteosarcoma 10, 11, 12, 13. Cyclooxygenase (COX), also called prostaglandin‐endoperoxide synthase (PTGS), is a key enzyme catalyzing the conversion of arachidonic acid to prostaglandin. COX has two isoforms: constitutive COX (COX‐1) and inducible COX (COX‐2) 14. The COX‐2 gene, encoding the inducible isozyme, is activated by growth factors, inflammatory stimuli, or carcinogenic factors, and its expression is usually undetectable in most normal tissues 15, 16. Furthermore, COX‐2 is involved in the regulation of cell proliferation and apoptosis, stimulation of angiogenesis, and invasion 17, 18, 19. Some studies suggest that COX‐2 expression may correlate with the development and progression of several types of human malignancies 20, 21, 22. The expression of COX‐2 is related to an increased risk of skin cancer 23, as well as to tumor size, lymph node metastasis, and poor prognosis in breast 24 and colorectal and ovarian cancers 25, 26. Nevertheless, no significant role of the expression of COX‐2 was found on the survival of patients with non‐small‐cell lung cancer 27. Importantly, COX‐2 may regulate the genesis and progression of osteosarcoma 28 and COX‐2 expression has been reported in patients with osteosarcoma 29, 30, 31, 32. In earlier studies, the percentage of osteosarcoma patients with COX‐2‐positive expression ranged from 16.7% to 81.2% 33, 34. Therefore, further investigations on the clinical significance of COX‐2 expression in osteosarcoma patients are required.

Benign bone tumors include osteochondroma, osteoma, osteoid osteoma, osteoblastoma, giant cell tumor, aneurysmal bone cyst, fibrous dysplasia, and enchondroma 35. Osteochondroma is the most common nonmalignant bone tumor, accounting for approximately 35% of all benign bone tumors 35. However, the malignant transformation into osteosarcoma has not been sufficiently studied, and its mechanism is still unclear 36. The sample sizes of the individual studies were generally small 29, 30, 31, 32, 33, 34, which might have led to a lack of power in the statistical analysis.

In this study, the present meta‐analysis integrated all available publications in a larger population to evaluate whether COX‐2 expression is associated with an increased risk of osteosarcoma in a comparison between osteosarcoma and benign osteochondroma cases. Additionally, we analyzed the possible clinicopathological and prognostic significance of COX‐2 expression in patients with osteosarcoma.

## Materials and methods

### Search strategy

A systematic search was performed of different electronic databases, including PubMed, Embase, EBSCO, Wanfang, and CNKI, for eligible papers published before June 2017. The following key words and search terms were used: (COX‐2 OR COX2 OR Cyclooxygenase‐2 OR PTGS2 OR Prostaglandin Synthase) AND (expression OR expressed) AND (osteosarcoma OR osteogenic sarcoma). Moreover, the reference lists of the included publications were also retrieved to find other potentially relevant studies.

### Selection criteria

The following inclusion criteria were applied in this meta‐analysis: (a) confirmation of osteosarcoma by histopathological examination; (b) COX‐2 protein expression tissue analyses using immunohistochemistry (IHC); (c) studies with complete information concerning the rate of COX‐2 protein expression in malignant and benign osteochondroma; (d) studies with sufficient information to assess the correlation of COX‐2 expression with clinical characteristics of patients with osteosarcoma; and (e) studies providing sufficient information to evaluate the prognostic effect of COX‐2 expression in osteosarcoma by multivariate analysis. Only the complete publications with more extensive information or larger populations were included when the authors had published more than one article using overlapping study populations.

### Data extraction and study selection

The following data were extracted from the eligible publications: the last name of the first author; year of publication; country; ethnicity; case number (osteosarcoma and benign osteochondroma); immunohistochemical staining patterns; cutoff values; median or mean years; tumor stage; frequency of COX‐2 expression; clinical characteristics, such as age (≥20 years vs. ≤20 years), gender (male vs. female), tumor location (femur vs. nonfemur), cancer histology (osteogenic osteosarcoma vs. nonosteogenic osteosarcoma), tumor grade (grade 3–4 vs. grade 1–2), clinical stage (stage 3–4 vs. stage 1–2), necrosis (≥90% vs. <90%), and metastasis (yes vs. no); and the prognosis from multivariate analysis. We analyzed data from 2 × 2 tables. The quality of the eligible studies was in accordance with the guidelines of the Cochrane Collaboration and the Preferred Reporting Items for Systematic Reviews and Meta‐Analyses (PRISMA) statement criteria 37. A total number of 391 publications were found: 83 records in PubMed, 96 records in Embase, 77 records in EBSCO, 75 records in Wanfang, and 60 records in CNKI. Final 23 studies were identified in this meta‐analysis. To categorize a patient as COX‐2‐positive or COX‐2‐negative, the COX‐2 expression, determined using IHC staining, was considered positive or negative based on the cutoff values of the original articles.

### Statistical analysis

Data analysis was performed using the stata software (version 12.0; Stata Corporation, College Station, TX, USA). The combined odds ratios (ORs) and their 95% confidence intervals (95% CIs) were calculated to estimate the relationships of COX‐2 expression in osteosarcoma and benign osteochondroma. The correlations of COX‐2 expression with the clinical characteristics of osteosarcoma patients were also analyzed using the overall ORs and their 95% CIs. The overall hazard ratios (HRs) with their 95% CIs were determined to evaluate the prognostic effect of COX‐2 expression in osteosarcoma patients for multivariate analysis. Heterogeneity among the studies was detected using the Cochran's Q statistic 38. The random‐effects model was used in this meta‐analysis. Subgroup, meta‐regression, and sensitivity analyses were conducted of the results with substantial heterogeneity (*P *<* *0.1), to explain the potential sources of heterogeneity 39, 40. The possible publication bias was measured using Egger's test in more than eight studies 41.

### Trial sequential analysis

Trial sequential analysis (TSA) was performed to reduce type I error and to assess the required information size for determination of the statistical significance 42, 43. In the meta‐analysis, the type I and type II errors were considered to be 5% and 20%, respectively. The relative risk reduction (RRR) was set to be of 20% for the outcome, and a statistical test power of 80% was defined. The cumulative *Z*‐curve crossed the trial sequential monitoring boundary or the required study population information, which suggested that the conclusion drawn in the meta‐analysis was positive; otherwise, more studies with larger sample sizes were indicated to be necessary for consistency in the evidence 44, 45.

## Results

### Characteristics of the relevant publications

**Figure 1:**
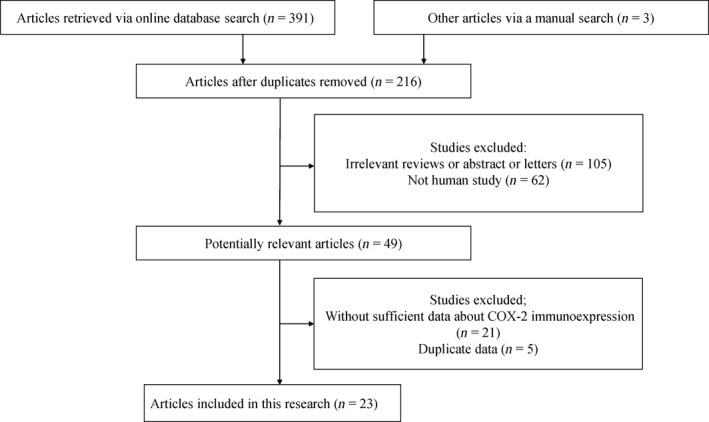
Flow diagram of the study selection.

Figure 1 illustrates the above‐described search strategy. After the search of the relevant databases, 23 studies with a total of 1084 patients with osteosarcoma and 247 patients with benign osteochondroma were eventually included in the current meta‐analysis. All these studies published from 2003 to 2016 had used IHC analysis of COX‐2 expression 29, 30, 31, 32, 33, 34, 46, 47, 48, 49, 50, 51, 52, 53, 54, 55, 56, 57, 58, 59, 60, 61, 62. Eleven of these eligible studies compared the expression of COX‐2 between osteosarcoma and benign osteochondroma. The associations between COX‐2 expression and the clinical features of patients with osteosarcoma were examined in 21 studies. Two original studies reported the prognostic role of COX‐2 expression using multivariate analysis in patients with osteosarcoma. The general characteristics of the eligible papers are listed in Table 1 and Table 2.

**Table 1:** Basic characteristics of the included publications. E+, positive expression status; IHC, immunohistochemistry; MA, multivariate analysis; N, study population; NA, not applicable

First author	Country	Ethnicity	Age	Stage	Location	Osteosarcoma	Osteochondroma	Clinical features	MA‐survival	IHC cutoff (positivity)
						N (E+ %)	N (E+ %)			
Dickens 46	USA	Caucasians	NA	NA	Cytoplasm	99 (66.7)		Yes	No	25%
Jiang 47	China	Asians	NA	NA	Cytoplasm	31 (67.7)		Yes	No	5%
Zhu 48	China	Asians	25.5	NA	Cytoplasm	31 (61.3)	30 (6.7)	Yes	No	5%
Hosono 34	Japan	Asians	29	NA	Cytoplasm	30 (16.7)		Yes	No	10%
Masi 33	Italy	Caucasians	19	NA	Cytoplasm	42 (59.5)		Yes	No	20%
Ou 49	China	Asians	18.6	1–3	Cytoplasm	49 (77.6)	20 (35)	Yes	No	5%
Wang 50	China	Asians	18.68	NA	Cytoplasm	40 (67.5)	20 (15)	No	No	5%
Rodriguez 32	USA	Mix	17	NA	NA	36 (47.2)		Yes	No	10%
Geng 51	China	Asians	19.3	1–3	Cytoplasm	59 (69.5)		Yes	No	10%
Zhan 52	China	Asians	19	1–3	Cytoplasm	38 (57.9)	20 (25)	Yes	No	5%
Urakawa 31	Japan	Asians	15	2	NA	51 (23.5)		Yes	Yes	80%
Liu 53	China	Asians	21.8	2–3	Cytoplasm	50 (70)	20 (30)	Yes	No	5%
Liao 54	China	Asians	42.1	1–3	Cytoplasm	57 (70.2)	18 (11.1)	Yes	No	10%
Huang 55	China	Asians	NA	1–3	Cytoplasm	37 (86.5)		Yes	No	30%
Boulytcheva 30	Russia	Caucasians	NA	1–4	Cytoplasm	34 (32.4)		Yes	Yes	10%
Li 56	China	Asians	19	1–3	Cytoplasm	85 (62.4)	20 (15)	Yes	No	10%
Xu 57	China	Asians	20	1–3	Cytoplasm	28 (78.6)	25 (36)	Yes	No	5%
Ma 58	China	Asians	NA	1–3	Cytoplasm	45 (80)		Yes	No	10%
Duan 29	China	Asians	NA	NA	Cytoplasm	30 (73.3)	20 (25)	Yes	No	0%
Chen 59	China	Asians	18.5	2–3	Cytoplasm	49 (49)		Yes	No	10%
Meng 60	China	Asians	NA	NA	Cytoplasm	52 (71.2)	40 (35)	No	No	10%
Lian 61	China	Asians	21.1	2–3	Cytoplasm	35 (82.9)	14 (14.3)	Yes	No	0%
Zhu 62	China	Asians	27.5	NA	Cytoplasm	76 (68.4)		Yes	No	5%

**Table 2:** Basic characteristics of the eligible studies with clinical characteristics

First author	>/= 20 years	</= 20 years	Male	Female	Femur	Nonfemur	Osteogenic OS	Nonosteogenic OS	Grade 3–4	Grade 1–2	Stage 3–4	Stage 1–2	Necrosis	No	Metastasis	No
	E/N	E/N	E/N	E/N	E/N	E/N	E/N	E/N	E/N	E/N	E/N	E/N	E/N	E/N	E/N	E/N
Dickens 46			11/24	15/21									6/9	8/18	24/32	23/35
Jiang 47															14/17	7/14
Zhu 48			13/22	6/9					9/10	10/21					14/17	5/14
Hosono 34	1/16	4/14	4/16	1/14	4/18	1/12	0/16	5/14	5/26	0/4						
Masi 33									23/32	2/10						
Ou 49							15/19	23/30			12/13	26/36			12/13	26/36
Rodriguez 32			7/8	3/7	9/21	8/15	6/8	11/28					7/14	10/22	6/10	11/26
Geng 51							23/35	18/24							7/8	34/51
Zhan 52											8/11	14/27				
Urakawa 31	5/20	7/31	9/33	3/18									7/18	5/33	8/18	4/33
Liu 53											14/15	21/35				
Liao 54							15/22	25/35			22/23	18/34				
Huang 55							12/14	19/23			10/11	22/26			15/16	17/21
Boulytcheva 30															6/11	5/23
Li 56									25/31	28/54	21/25	32/60			36/48	17/37
Xu 57							11/14	11/14			8/8	14/20				
Ma 58			29/37	7/8			23/31	12/14			12/13	24/32				
Duan 29							9/13	13/17	7/9	15/21						
Chen 59													12/29	12/20		
Lian 61			18/22	11/13							12/12	17/23			22/24	7/11
Zhu 62									23/25	29/51						

E, positive expression status; N, study population; NA, not applicable; OS, osteosarcoma.

### Association between COX‐2 expression and osteosarcoma development

**Figure 2:**
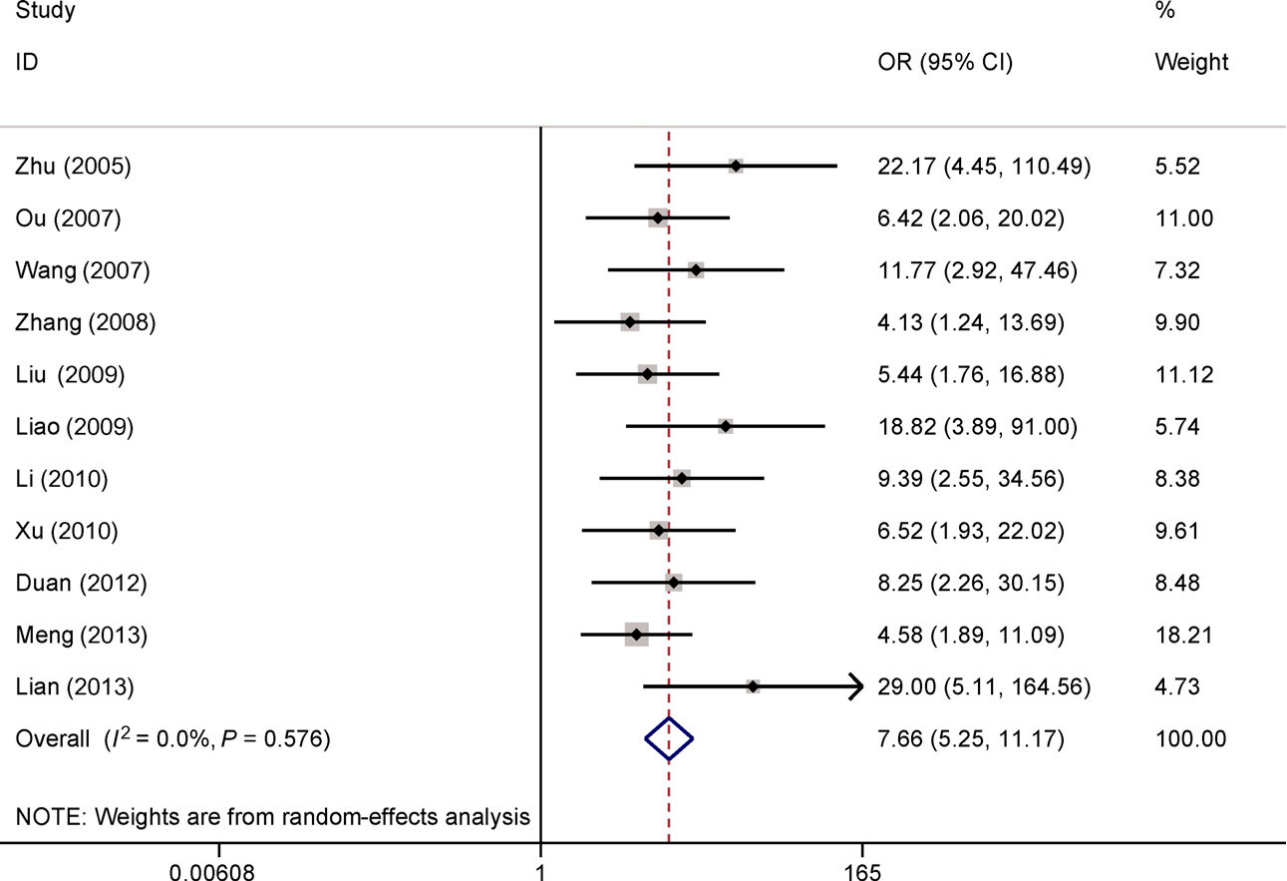
Forest plot of the association of COX‐2 immunoexpression between osteosarcoma and benign osteochondroma (OR = 7.66, *P *<* *0.001).

The data from 11 comparative studies, including of 495 patients with osteosarcoma vs. 247 benign osteochondroma patients, revealed that COX‐2 expression was significantly more increased in osteosarcoma than in benign osteochondroma (OR = 7.66, 95% CI = 5.25–11.17, *P *<* *0.001) (Fig. 2).

### Association of COX‐2 expression with tumor grade and clinical stage in osteosarcoma

**Figure 3:**
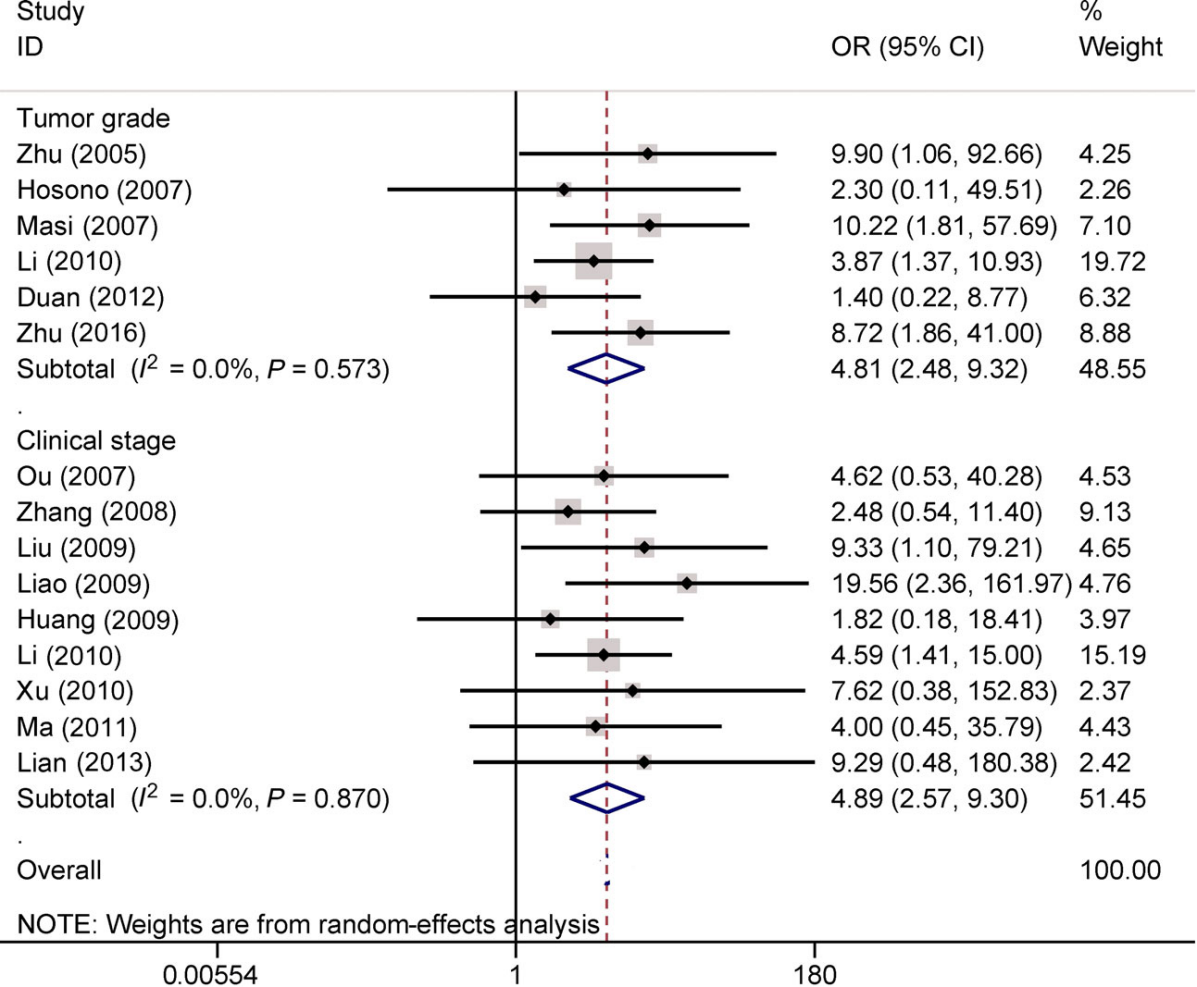
Forest plot of the correlation of COX‐2 immunoexpression with the tumor grade (OR = 4.81, *P *<* *0.001) and clinical stage (OR = 4.89, *P *<* *0.001).

Six studies with 294 osteosarcoma patients found a significant relationship between COX‐2 expression and tumor grade (high grade vs. low grade: OR = 4.81, 95% CI = 2.48–9.32, *P *<* *0.001) (Fig. 3).

Additionally, nine studies with 424 osteosarcoma patients discovered a significant correlation was observed between COX‐2 expression and clinical stage (stage 3–4 vs. stage 1–2: OR = 4.89, 95% CI = 2.57–9.30, *P *<* *0.001) (Fig. 3).

### Association of COX‐2 expression with metastasis in osteosarcoma

**Figure 4:**
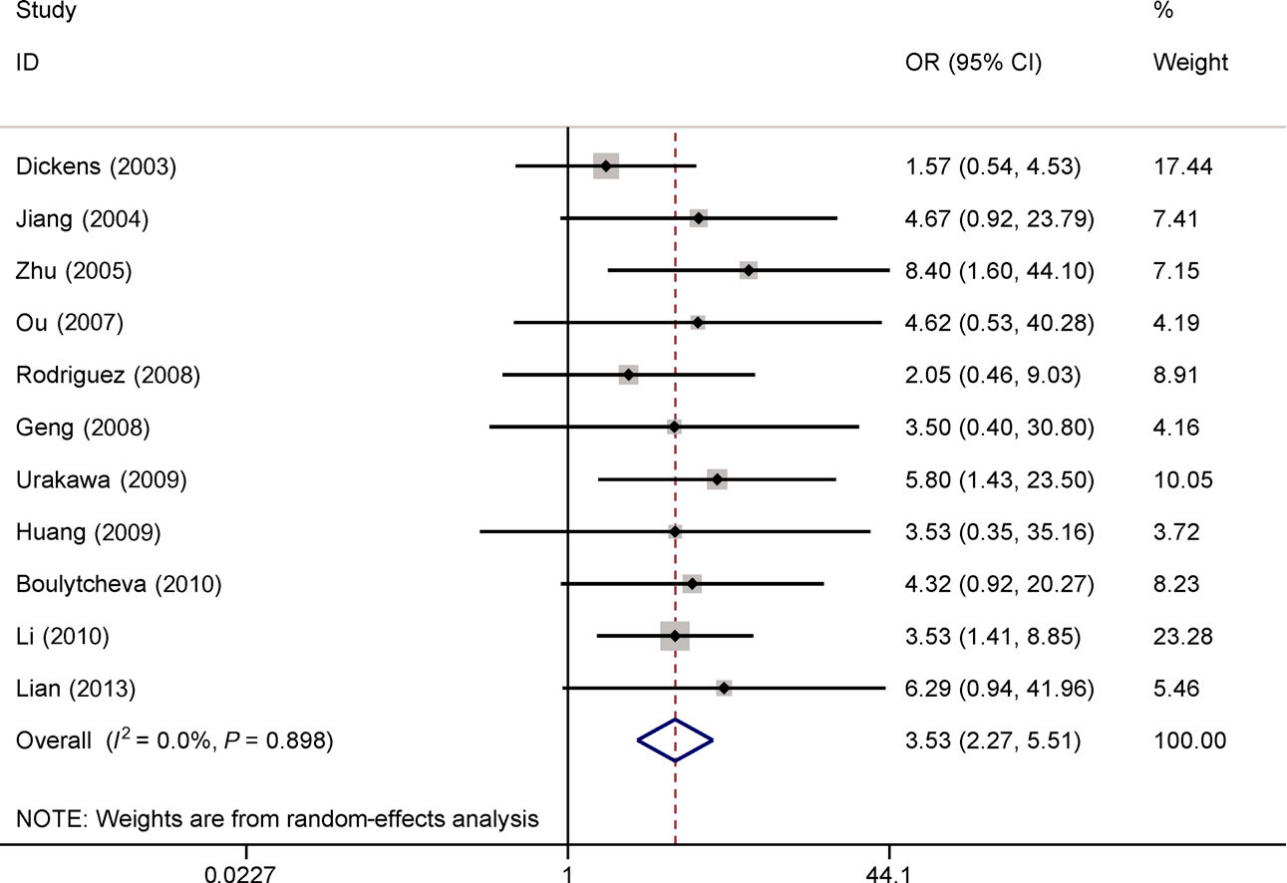
Forest plot of the correlation of COX‐2 immunoexpression with metastasis (OR = 3.53, *P *<* *0.001).

The results obtained in 11 studies with 515 osteosarcoma patients demonstrated that COX‐2 expression was significantly associated with metastasis in osteosarcoma (OR = 3.53, 95% CI = 2.27–5.51, *P *<* *0.001) (Fig. 4).

### Prognostic role of COX‐2 expression for multivariate analysis in osteosarcoma

Cyclooxygenase‐2 expression was linked to worse metastasis‐free survival (*P *=* *0.029) in 51 osteosarcoma patients 31. Moreover, Boulytcheva *et al*. 30 reported that COX‐2 expression in 34 patients with osteosarcoma was associated with poor overall survival and relapse‐free survival (*P *<* *0.05). Nonetheless, additional studies are needed to further validate the findings concerning the prognostic effect of COX‐2 expression established by multivariate survival analysis in osteosarcoma.

### Heterogeneity and publication bias

**Figure 5:**
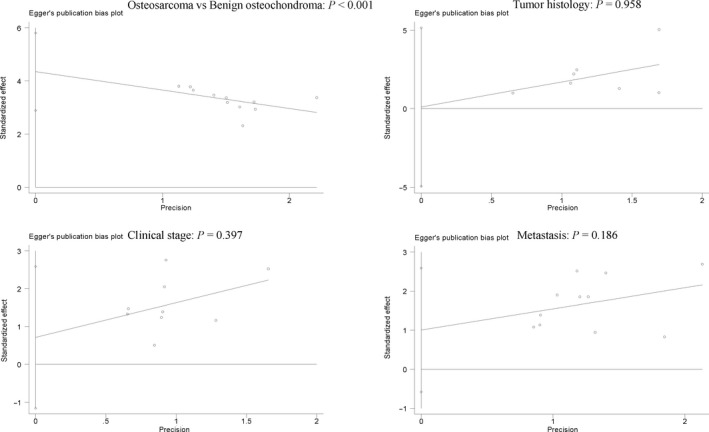
Forest plot of the possible publication bias determined using Egger's test (osteosarcoma vs. benign osteochondroma: *P *<* *0.001), cancer histology (*P *=* *0.958), clinical stage (*P *=* *0.397), and metastasis (*P *=* *0.186).

**Figure 6:**
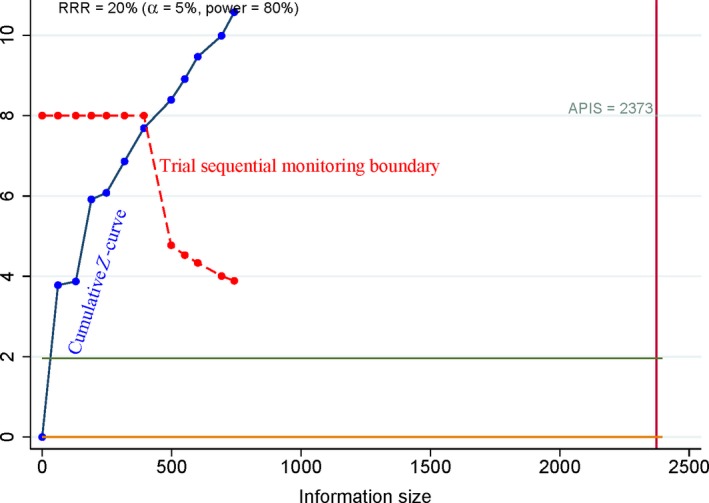
Trial sequential analysis of COX‐2 immunoexpression in osteosarcoma vs. benign osteochondroma. The cumulative Z‐curve crossed the trial sequential monitoring boundary, suggesting that the result was conclusive.

No significant heterogeneity was detected in our meta‐analysis (all *Ps *> 0.1) (Figs 2, 3, 4, 5, 6).

Egger's test was applied to measure the publication bias, and significant heterogeneity was found in the comparison of osteosarcoma and benign osteochondroma (*P *<* *0.001) (Fig. 5). However, no evidence of publication bias was found between COX‐2 expression and metastasis, clinical stage, or cancer histology in osteosarcoma (*P *>* *0.05) (Fig. 5).

### Trial sequential analysis

**Figure 7:**
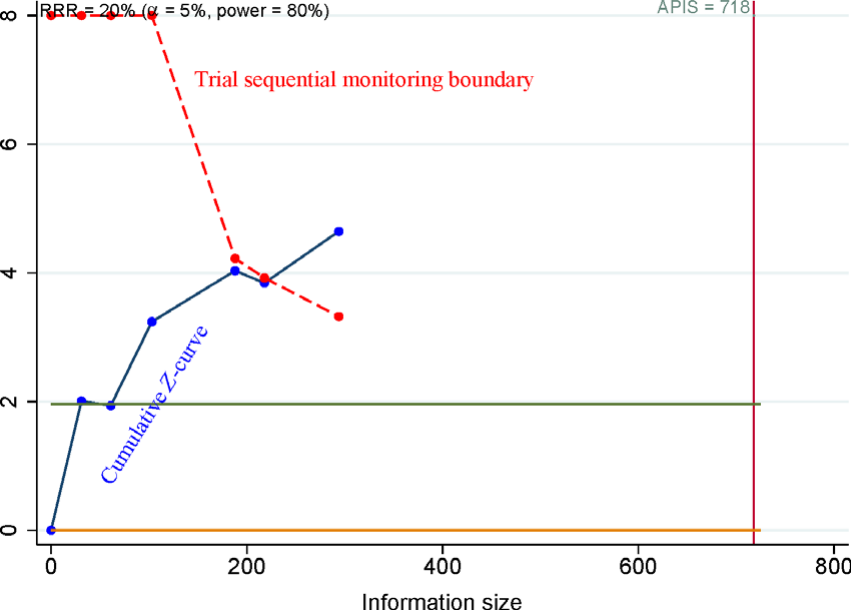
Trial sequential analysis of COX‐2 immunoexpression in relation to tumor grade. The cumulative *Z*‐curve crossed the trial sequential monitoring boundary, suggesting that the result was reliable.

**Figure 8:**
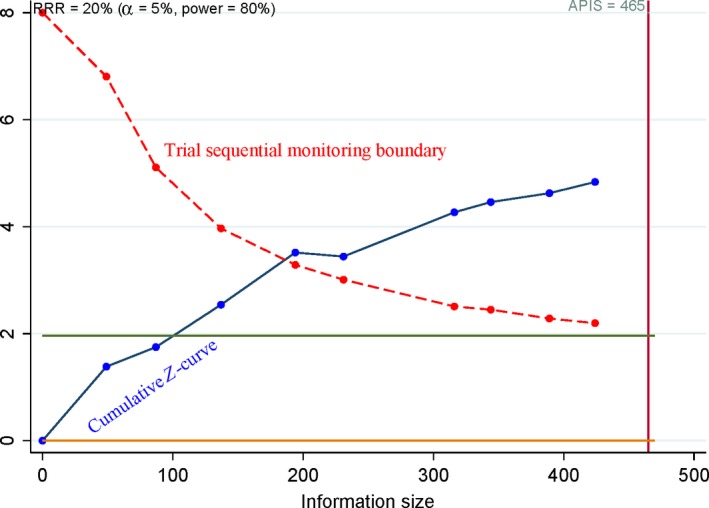
Trial sequential analysis of COX‐2 immunoexpression in relation to clinical stage. The cumulative Z‐curve crossed the trial sequential monitoring boundary, suggesting that the result was reliable.

**Figure 9:**
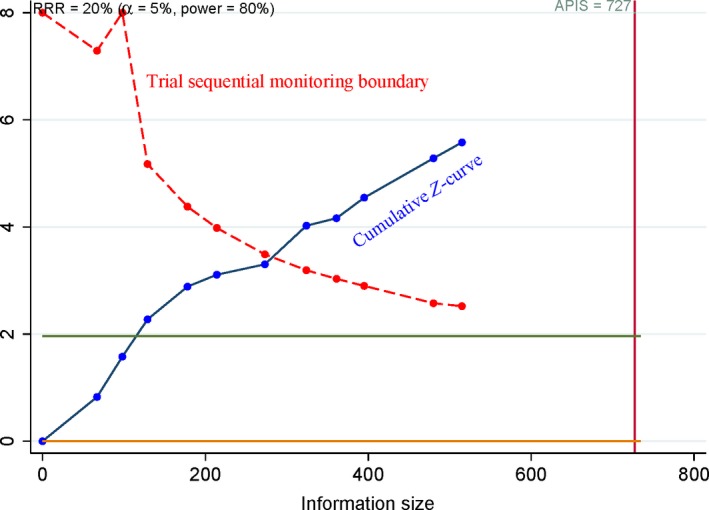
Trial sequential analysis of COX‐2 immunoexpression in relation to metastasis. The cumulative Z‐curve crossed the trial sequential monitoring boundary, suggesting that the result was conclusive.

Based on the a priori anticipated information size (APIS) method, when osteosarcoma was compared to benign osteochondroma (Fig. 6), the patients with high‐grade cancer were compared to patients with low‐grade cancer (Fig. 7), advanced‐stage patients were compared to early‐stage patients (Fig. 8), and the patients with metastasis were compared to patients without metastasis (Fig. 9). The cumulative *Z*‐curve crossed the trial sequential monitoring boundary for the above analyses, which suggested that these results were reliable and firm.

### Association of COX‐2 expression with age, gender, or tumor location in osteosarcoma

**Figure 10:**
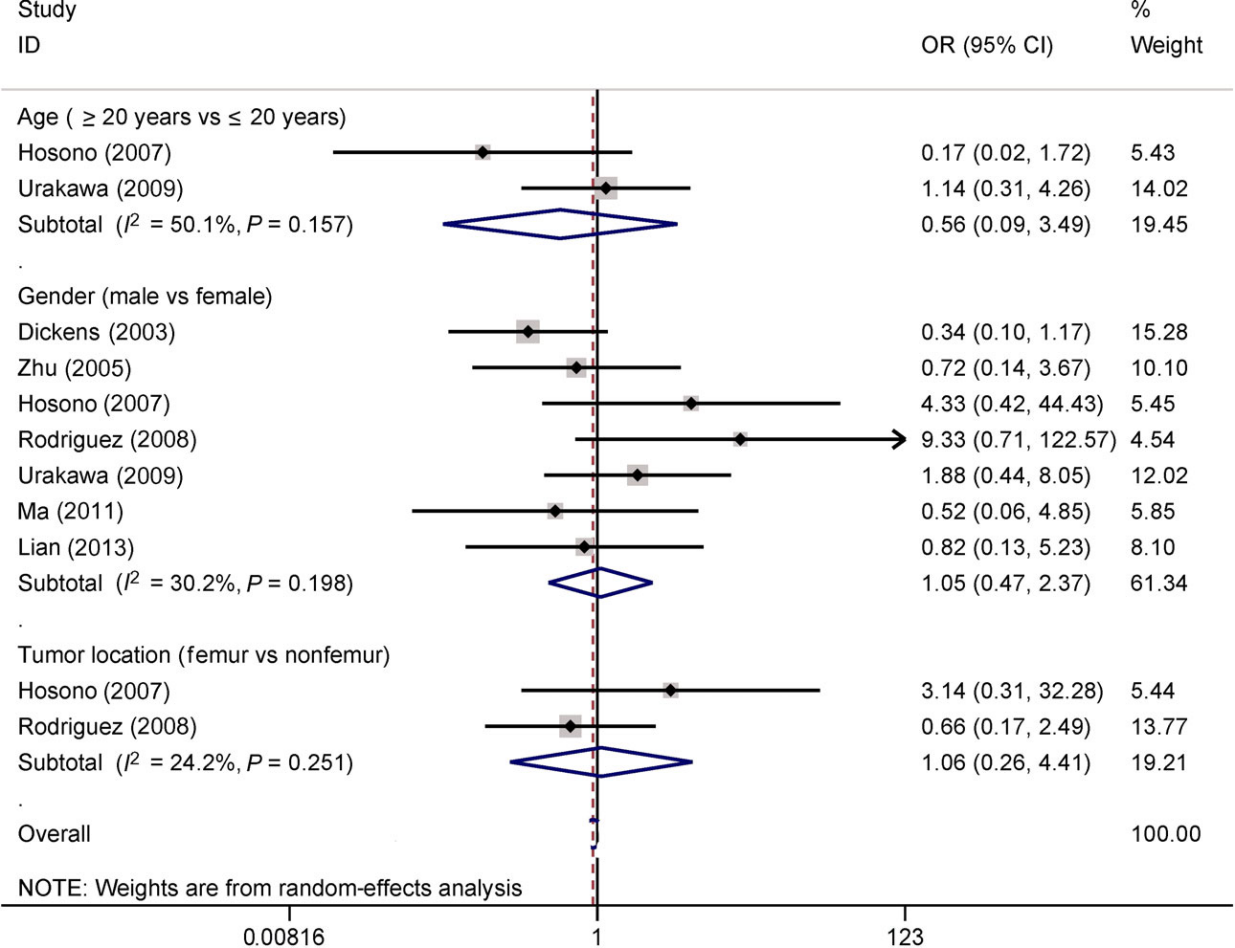
Forest plot of the association of COX‐2 immunoexpression with age factor, gender, and tumor location (*P *>* *0.1).

We explored whether the expression of COX‐2 was correlated with the clinical characteristics of patients with osteosarcoma. The results showed that COX‐2 expression was not associated with age (two studies with 81 cases: ≥ 20 years vs. ≤ 20 years), gender (seven studies with 252 cases: male vs. female), or tumor location (two studies with 66 cases: femur vs. nonfemur) of osteosarcoma (OR = 0.56, 95% CI = 0.09–3.49, *P *=* *0.534; OR = 1.05, 95% CI = 0.47–2.37, *P *=* *0.903; OR = 1.06, 95% CI = 0.26–4.41, *P *=* *0.933, respectively) (Fig. 10).

### Association of COX‐2 expression with histology and necrosis in osteosarcoma

**Figure 11:**
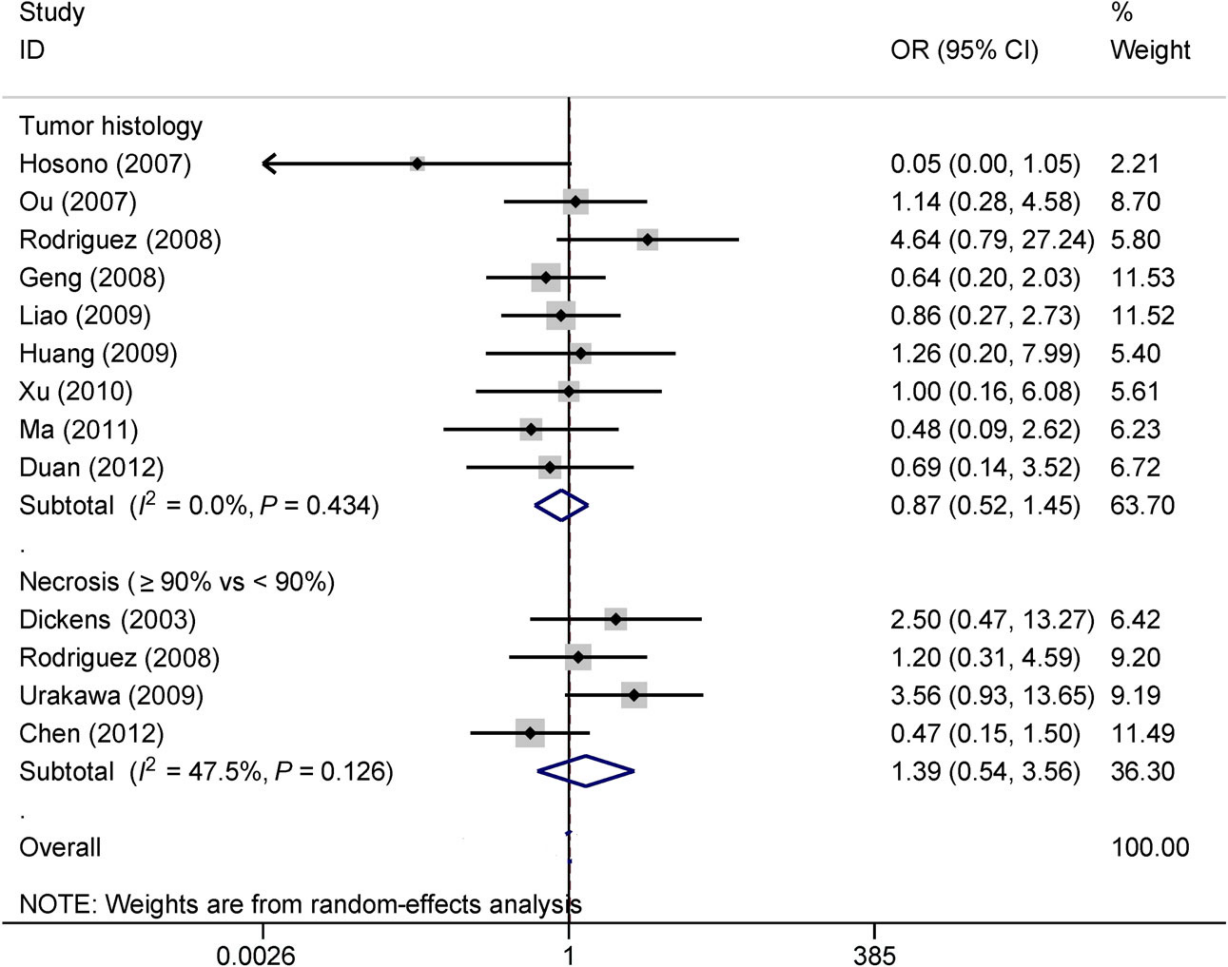
Forest plot of the correlation of COX‐2 immunoexpression with cancer histology and necrosis (*P *>* *0.1).

The results showed no significant correlation between COX‐2 expression and cancer histology (nine studies with 371 cases: osteogenic osteosarcoma vs. nonosteogenic osteosarcoma: OR = 0.87, 95% CI = 0.52–1.45, *P *=* *0.583) (Fig. 11) or necrosis (four studies with 163 cases: ≥ 90% vs. < 90%: OR = 1.39, 95% CI = 0.54–3.56, *P *=* *0.491) (Fig. 11).

## Discussion

Using the IHC method, the authors of a number of earlier studies have demonstrated that COX‐2 is frequently expressed in a variety of human cancers, including lung cancer 30, breast carcinoma 20, colorectal cancer 63, and nasopharyngeal carcinoma 64. COX‐2 immunoexpression was also detected in osteosarcoma 29, 30. The meta‐analysis of Jiao *et al*. 65 involving 14 studies reported that COX‐2 expression was correlated with the clinical stage and metastasis of osteosarcoma. However, the meta‐analysis of Wang *et al*. involving nine studies established that COX‐2 expression was not associated with the metastasis and clinical stage of osteosarcoma 66. To compare whether COX‐2 expression was different in osteosarcoma and benign osteochondroma, the results from 11 studies (495 osteosarcomas vs. 247 benign osteochondromas) demonstrated that COX‐2 expression in osteosarcoma was notably higher than in benign osteochondroma (OR = 7.66, *P *<* *0.001), which suggested that COX‐2 expression as a potential marker using the IHC method could distinguish osteosarcoma and benign osteochondroma. TSA revealed that true‐positive results were obtained in the comparative analysis of osteosarcoma and benign osteochondroma.

On further evaluation, the relationship between COX‐2 expression and the clinical characteristics of patients with osteosarcoma showed no significant correlation of COX‐2 expression with age, gender, tumor location, cancer histology, and necrosis across each study (Figs 10 and 11). And these results from all pooled studies remained constant. COX‐2 expression was not associated with tumor grade in osteosarcoma 29, 34, but Masi *et al*. 33 reported that COX‐2 expression was correlated with tumor grade. Pooled data from six studies with 294 cases revealed that COX‐2 expression was positively linked to tumor grade (OR = 4.81) and clinical stage (OR = 4.89). Dickens *et al*. 46 found no significant association between COX‐2 expression and metastatic status. Rodriguez 2008 *et al*. also reported that no correlation existed between COX‐2 expression and metastasis in osteosarcoma 32. Conversely, Urakawa *et al*. 31 reported that the expression of COX‐2 was significantly related to the metastatic status of osteosarcoma. Further analysis from 11 studies with 515 osteosarcoma patients revealed the presence of a positive association between COX‐2 expression and the metastatic status of osteosarcoma in a larger population. The above‐mentioned analyses suggest that the expression of COX‐2 may play an important role in disease progression and metastasis in patients with osteosarcoma. No evidence of substantial heterogeneity was available concerning the clinical features. Further TSA revealed that future additional studies are not essential. Therefore, the results of our analysis are conclusive.

The multivariate analysis showed that COX‐2 expression was associated with worse prognosis in metastasis‐free survival (5 years) 31, overall survival, and relapse‐free survival 30, which indicated that COX‐2 expression might become a potential prognostic marker. Additional clinical research of larger sample sizes using multivariate analysis in patients with osteosarcoma is necessary to confirm the prognostic significance of COX‐2 expression.

The present meta‐analysis has several limitations. First, the patients included in the analyses were mainly Asians and Caucasians, but the shares of other ethnic groups, such as the African population, were insufficient. Second, publication bias was detected in the comparison of osteosarcoma and benign osteochondroma. Papers with positive conclusions were more easily published than articles with negative conclusions. In addition, publications of other styles, such as conference abstracts, were lacking due to insufficient information. Third, more studies using multivariate analysis of COX‐2 expression are needed to confirm the prognostic effect in different ethnic groups. Finally, the cutoff values of COX‐2 expression of the eligible studies were different. Thus, in the future, whether COX‐2 expression is positive or negative should be defined based on common standards set.

In conclusion, the present findings suggest that COX‐2 immunoexpression is significantly higher in osteosarcoma than in benign osteochondroma. Additionally, COX‐2 expression is associated with tumor grade, clinical stage, and metastasis of osteosarcoma but is not correlated with age, gender, tumor location, histology, or necrosis. The expression of COX‐2 may serve as a prognostic indicator for the multivariate analysis of metastasis‐free, overall, and relapse‐free survival. Conducting additional, well‐designed, prospective studies that investigate large populations in the future is essential to further validate the prognostic role of COX‐2 expression.

## Author contributions

ZG contributed to the conception and design of this study. SW, HG, and JZ contributed to acquisition, analysis, and interpretation of data, and drafting and revising the article. All authors gave final approval of the version to be published and agreed to be accountable for all aspects of the work in ensuring that questions related to the accuracy or integrity of any part of the work are appropriately investigated and resolved.

## Conflict of interest

The authors declare no conflict of interest.
